# COVID-19 Resilience in the Third Sector

**DOI:** 10.1177/11786329211013547

**Published:** 2021-04-30

**Authors:** Sonia Cottom

**Affiliations:** Pain Association Scotland, York St John University York Business School, York, UK

**Keywords:** Chronic Pain, Self-Management, COVID-19

## Abstract

Chronic pain is a major clinical challenge in Scotland and across Europe as a whole. 18% of the UK population are currently affected by severe chronic pain. This has resulted in a significant impact on people’s quality of life and affects their family, relationships and carers. This article discusses how a third sector organisation – Pain Association Scotland (PAS) – has completely changed their approach to service delivery after 33 years as a result of the COVID-19 pandemic as well as how they have worked with Health Boards in order to ensure a continued service which is equitable, trusted and provides continuity of service. The discussions are from the perspective of the Director, Sonia Cottom, BA (Hons) who has been with the Association for 10 years and who is ultimately responsible for the operations, strategic planning and service improvement and implementation. She is also advisor to the Scottish Government on chronic pain policy, providing the evidence to underpin future strategic decision making.

## Introduction

Chronic pain is a major clinical challenge in Scotland and across Europe as a whole. About 18% of the UK population are currently affected by severe chronic pain.^1^ This has resulted in a significant impact on people’s quality of life and affects their family, relationships and carers.

This article discusses how a third sector organisation – Pain Association Scotland (PAS) – has completely changed their approach to service delivery after 33 years as a result of the COVID-19 pandemic as well as how they have worked with Health Boards in order to ensure a continued service which is equitable, trusted and provides continuity of service. The discussions are from the perspective of the Director, Sonia Cottom, BA (Hons) who has been with the Association for 10 years and who is ultimately responsible for the operations, strategic planning and service improvement and implementation. She is also advisor to the Scottish Government on chronic pain policy, providing the evidence to underpin future strategic decision making. The views formed are from the working practices, the Associations’ in-house evaluation data and the wider context of chronic pain throughout Scotland.

## Background

Pain Association Scotland supports people suffering with the effects of living with chronic pain as a long-term condition either in its own right or as a result of another underlying condition.

They do this by providing professional self-management training which extends to carers, family members and veterans via one-to-one sessions and also using digital technology (Attend Anywhere) for those who would otherwise be denied a service. They continue to address the non-medical and psychological issues associated with chronic pain and the nature of our interface between healthcare, family and community means that innovating new ways of working is a significant feature of our service. With over 33 years’ experience of working with this population of people, clinicians, social workers, carers, friends and employers, it has enabled the Association to pioneer and develop a highly interactive learning and support model which is adaptable to the needs in the different geographical areas we serve throughout Scotland. Chronic pain requires significant help and time in both clinical and non-clinical settings especially as patients use primary care services 5 times more than any other condition with a related cost of almost £10 billion to the UK economy.^2^

Their self-management model is a professionally led service, developed and delivered over the years by staff who understand the issues and complex nature of living with chronic pain. It consists of a rolling programme of monthly group meetings and as well as intensive 5 week courses with the aim of providing service users with valuable tools to help them lead an improved quality of life. The number of groups and courses provided within the various Scottish Health Boards is dependent on the needs of each respective Health Board of Integrated Joint Board. From this, the Associations’ services are commissioned through Service Level Agreements (SLA’s) with the respective Boards each year.

Chronic pain has a high impact upon, physical, psychological and family health. Significant issues include: depression, long term stress, isolation, high levels of medication, poor mobility, lack of self-esteem and fatigue.^3^ On average each year they provide 1166 hours of face-to-face self-management training within a group setting in the community for approximately 3800 people. The service is set-up in a way that maximises access for patients who can self-refer as well as referring clinicians – the referral criteria is an important issue to consider in the context of access, because many patients are excluded from clinical pain management programmes due to a strict/rigorous screening process. In the context of clinicians referring patients directly to PAS for self-management education and training, this has usually been done at the end of a patient’s journey when all medical investigations and interventions have been explore and there is basically nothing more medically that can be done for the patient. PAS for a number of years have been campaigning for people to be guided and referred for self-management at the very start of their pain pathway in order that they can begin to adopt and implement a series of self-help strategies to help them through their medical journey.

It is during this COVID-19 crisis that there has been an increased demand for the services of PAS which very quickly had to move to on-line delivery – in Scotland chronic pain sufferers have been faced with the following scenario which naturally compounds their daily issues of heightened stress, anxiety and suffering:

✓ 11 out of 14 Chronic Pain services have ceased all new patient activity due to staff redeployment. This means that there will be no first appointments at a chronic pain clinic for those referred so they will remain on a waiting list. Compare this to pre-pandemic when there were very few patients in any of the Health Boards waiting more than 18 weeks for access to a first appointment.
✓ There are no virtual support groups being delivered by the NHS within the Pain Management Service.
✓ All procedures and interventions had ceased.
✓ Service provision varies greatly as a result of capacity therefore putting patients at risk of being in a postcode lottery.
✓ Chronic pain services are fearful once again during this second wave and second lockdown of increased demands as the situation continues and are already starting to withdraw services once again.

In response to such issues, PAS continue to work in collaboration with various NHS chronic pain services throughout Scotland. The situation needed to be mindful of for the future is the increased demand to the pain services as a result of the 2 lockdowns and naturally their limited capacity when chronic pain services do resume along with the lengthy waiting lists. PAS have therefore been working with the Scottish Government to provide an on-line tool as a replacement for a first appointment at a chronic pain clinic so at least people can have some help with their chronic pain whom would otherwise be disadvantaged and having to endure lengthy waiting lists. This pathway then has the potential to help redirect people who in reality do not actually need that first physical appointment and would hopefully therefore help reduce future waiting lists.

The COVID-19 pandemic has resulted in impairments for many as a result of delayed treatments and appointments and we have seen the result of this being a decrease in psychological wellbeing. Their work therefore forms part of the public health approach to both prehabilitation and rehabilitation by directly addressing some key areas that help people feel safer in their life, more self-reliant, improve their locus of control and generally cope better. For many of the current service users of PAS, the COVID pandemic has been the straw that broke the camel’s back and therefore people have been asking them for something quick and instant on-line to help them integrate this into their daily living. They are also aware that going forward, long COVID and the pain that can go along with this is going to be an issue for people.

Unfortunately PAS’s access to the Attend Anywhere digital remote platform (used to reach those patients in NHS Western Isles and NHS Dumfries and Galloway who were unable to access the face-to-face group settings) had to be withdrawn at the start of April 2020 due to the fact that clinicians were needing this access as all their consultations were being conducted via video-conferences. This platform was sometimes used for people in the more remote and rural Highlands and Islands, for example, the Western Isles, who were unable to travel to a group session prior to COVID-19. However, they found that this platform was rarely used due to lack of sufficient internet speed. This provided PAS with an opportunity for the following:

- Switching to Zoom meant that they could enable more people to join a session as Attend Anywhere only permitted 5 people in total.
- The sessions were set up as password protected for security purposes.
- Each person who was referred to their service or who enquired was ‘triaged’ by the Training Officers at PAS over the phone first of all in order to help them set the scene for them and help them to manage their expectations.
- At the end of the Zoom sessions, each person was emailed supporting handouts.
- All the courses delivered in both the Western Isles and in Dumfries and Galloway (remote and rural areas) have been opened up to Island and Locality-wide respectively. This increased demand resulted in delivering 2 sessions for each and in Dumfries and Galloway, there was a session delivered solely for Healthcare Professionals in order to help them better support patients with chronic pain.

Delivering in this new way enabled PAS to identify other aspects of how to improve their service using on-line content and ensure that people had equity of service. Some of this included:

- Provided 1244 hours of staff-led self-management training – an additional 78 hours from the usual yearly average.
- Recorded a series of ‘fireside chats’ with some of the service users around what self-management means to them. The difference it’s made to their life and encouraging others to attend.
- Provided access to self-management courses and groups for 6851 people, almost double the amount in a usual year.
- Delivery of on-line information sessions for patients directly referred from a local GP surgery for their patients whom they would normally see on a regular basis for their chronic pain.

As a result of their additional work, the Association were asked to feed in to and be part of a number of committees and consultations, some of which included NICE/SIGN/RCGP COVID-19 rapid guideline on the ‘management of the long-term effects of COVID-19’, COVID-19 Remote Monitoring Service Governance Framework and the Pain Management Remote Healthcare Pathway Advisory Group. All of this additional work, commitment and participation was done using existing staff resource.

Ultimately, for any provision of service, and definitely within the healthcare sector, it is vital that both health professionals and potential funders acknowledge clear benefits of improved patient health and well-being, cost effectiveness which are overarched by the adoption of self-management strategies and model architecture. Individuals who have participated in intensive self-management programmes report increased energy, less pain, less dependence on others and improved mental health. In addition to long-term management, pain management services in the community can play an important role in screening, diagnosis, treatment, referral, education, prevention and signposting.^4^

In terms of evaluating the effectiveness of this change in service delivery, the Association reflected and focused on case studies and anecdotal comments as it was found attempting to do the usual series of evaluation on-line following the sessions is difficult with people either not returning their evaluations or not being able to sit with them and talk them through it if they are having difficulty. People were therefore asked to note what they have gained from attending the courses and sessions, some examples of which are:

*‘Thank you so much for the handouts. It was a great meeting today I really needed it. I look forward to joining you in the Oban group. It is such a relief to know that we will still be able to attend group as I am sure I said to you the last time this group has been a lifeline for me and I feel I would really struggle without it. I am so grateful that you are allowing us the opportunity to join. I also think the friendship group is a great idea too.’*

‘Thank you for the past several weeks. As a sufferer of chronic pain you have put into terms I understand, the cycle of pain, and introduced methods I can employ to break the cycle’.

‘The contents have given me a lot to think about and I am so grateful for being able to be part of these sessions – they have been a lifeline’.

‘Throughout the weeks, you have touched on a lot of my negative traits, which I now realise were leading to an amplification of my pain levels’.

‘One of your tools, for example, employing the simple method of changing one small thing, I have used, and been able to change my preconception of how much pain I am in which is a significant step forward’.

## The Future, What it is Has Taught and the Challenges

Looking at the principles and priorities of the remobilisation framework from the pandemic, the role of the third sector is clearly an important one as it creates the opportunity for NHS Boards and Integrated Joint Boards (IJB’s) to integrate the contribution and utilisation of the third sector into the prioritisation of maintaining capacity for Covid-19. Working in collaboration with many of the Boards, they have clearly recognised the importance of digital support and are willing to embrace a more blended model of service delivery as an integral pathway. Within such remobilisation plans, PAS appreciate that there is a need to alleviate the backlog of referrals for pain services and after all these years, this is certainly more than ever the perfect opportunity to get self-management integrated better into Primary Care so that people are offered this as an initial first step to helping people manage their pain rather than automatically being referred to Secondary Care and enduring waiting lists. Findings from PAS show many of those who would usually be considered most distant from the digital world, are really enthusiastic to engage when they have the appropriate support, which is tailored to their needs.

On the point of future planning and referrals, it is welcomed across third sector organisations that our increased work and quickly adapted services have been recognised. However, cognisance must be taken that in order to continue to provide this increased level of support for Health and Social Care Partnerships and adapt to varying needs, sustainable funding is needed for this.

NHS Boards should not be put in the position of funding the third sector through endowment funding. Many are providing vital collaborative services, making a clear, positive effect on outcomes. It is clearly written in many agreed principles of endowment funding that such grants should not be used for substituting core provision – of which the Scottish Government has committed to in the new framework for chronic pain service delivery – and furthermore, this is not a sustainable method of funding for long-term planning.

It also follows on from the above that by supporting co-production methodology, funding needs to be made available to third sector in order to effectively plan, deliver services and report on outcomes. Many trust funders will now not fund anything they consider to be a core statutory provision within NHS services. So one can see how third sector could potentially be disadvantaged from funding especially if engaging collaboratively with NHS Boards.

If the value of self-management and reducing people’s long-term reliance on specialist services and treatments which demonstrate low clinical efficacy is clear, then patients need help to be better engaged with the concept of self-management. It is appreciated that time is maybe limited to explain self-management and it is also recognise that many people might not absorb all the details during a GP appointment, but this is maybe an example of the type of action needed within future framework around pathways, language and a more holistic modelling approach.

In terms of Data as a Primary Driver, it would be welcomed for agreed key measured outcomes (not necessarily outputs) to be recognised for the effectiveness of third sector provision. In being introduced and dealing with new healthcare professionals enquiring about PAS’s services, there are regular questions around the ‘evidence-base’ for their work. Whilst PAS report on 3 recognised evaluation tools, the credibility for self-management would be greater enhanced if we had the data to understand for example, the reduction in secondary care referrals when self-management is introduced at the time of presenting in primary care, the reduction/effect on prescribing, increase in quality of life. . .. . .. . ..as well as the key powerful anecdotal comments being recognised. Moving the focus from simple wait times and incorporating ‘key difference’ data can surely provide a much better picture of the **difference** being made (or not) and the ability to identify more clearly where the gaps are in service provision. Having statistics to show that there is a 18 week waiting list for 400 for a first appointment does not help in identifying if all those people really need to be there and asking the question what is happening to people in the meantime whilst they wait?

The importance of the third sector having a service structure has been discussed. A sustainable service model which empowers chronic pain sufferers, their carers, family and colleagues can facilitate positive, practical changes leading to improved levels of coping, well-being and quality of life, without impacting on the already under resourced NHS services. It has also demonstrated how positive, adaptive coping mechanisms can ultimately lead to a better quality of life, but how the right funding and pathway infrastructure needs to be in place in order for this to happen.
